# Activation of the hedgehog pathway in chronic myelogeneous leukemia patients

**DOI:** 10.1186/1756-9966-30-8

**Published:** 2011-01-16

**Authors:** Bing Long, Huanling Zhu, Cuixia Zhu, Ting Liu, Wentong Meng

**Affiliations:** 1 Department of hematology, West China Hospital, Sichuan University. Key lab of Hematology of Sichuan Province. 37 Guoxue xiang St. Chengdu, Sichuan, 610041, China

## Abstract

**Background:**

Hedgehog (Hh) signaling pathway is involved in regulation of many tissues development and oncogenesis. Recently, Hh signaling has been identified as a required functional pathway for leukemia stem cells(LSCs), and loss of this pathway impairs leukemia progression.

**Objectives:**

The aim of this study was to determine the expression of Hedgehog signaling molecules in Chronic Myelogeneous Leukemia (CML) patients and normal people by semiquantitative polymerase chain reaction (PCR) and to correlate mRNA expression to patients' clinical data.

**Results:**

Here, we showed that Sonic hedgehog (Shh), Smoothened (Smo), and Gli1 genes of Hh signaling were significantly upregulated in CML patients when compared with normal people (P < 0.001). The levels of Shh, Smo mRNA in chronic phase of CML patients were obviously lower than that in blast crisis (p < 0.05). There were no significant differences of Shh, Ptch1, Smo, Gli1 mRNA expression found when comparing CML patients of chronic phase(CP) with imatinib(IM) treated or not(p > 0.05).

**Conclusions:**

These findings suggested that activation of the Hh pathway maybe associated with CML progression. Treatment of CML with imatinib, a selective inhibitor of the BCR-ABL tyrosine kinase inhibitor, has no significant influence on the inhibition of Hh pathway of CML-CP patients.

## Introduction

Chronic myelogeneous leukemia (CML) is a clonal disease that originates from a single transformed hematopoietic stem cell (HSC) or multipotent progenitor cell harboring a chromosomal translocation between chromosome 9 and 22 [t(9;22)(q34;q11)], resulting in the formation of Philadelphia(Ph) chromosome and at the molecular level, a chimeric gene known as BCR-ABL responsible for CML initiation. CML often initiates in a chronic phase, and without intervention, eventually progresses to a terminal blastic phase. The introduction of imatinib mesylate, has revolutionized the disease management. However, imatinib does not cure CML, and one of the reasons is that imatinib does not kill leukemia stem cells (LSCs) in CML [1,2]. Recent studies suggest that developmental pathway like Hedgehog signaling pathway played a role during the expansion of BCR-ABL-positive leukemic stem cells [3,4]. Hedgehog ligands (Sonic hedgehog [Shh], Indian hedgehog [Ihh], and Desert hedgehog [Dhh]) produced by stroma cells bind to the seven-transmembrane domain receptor Patched (Ptch), thereby alleviating patched-mediated suppression of smoothened (Smo), a putative seventransmembrane protein. This results in a conformational change of Smo and subsequent activation of the pathway, leading to induction of the Gli transcription factors and transcription of target genes like Ptch1, cyclin D1, and Bcl2 [5-7]. This study shows the expression and significance of Hh signaling pathway target genes Shh, Ptch1, Smo and Gli1 in patients with CML.

## Materials and methods

### Samples

Sixty cases of CML treated at West China Hospital of Sichuan University were included in this study from May 2009 to January 2010.The diagnosis of CML was established on the basis of WHO Guideline. The positive results of both cytogenetic evaluation of *t*(9;22) and molecular study of BCR-ABL are required for the diagnosis. According to the WHO classification, CML patients were divided into three groups: chronic phase (CP), accelerated phase (AP) and blast crisis (BC). In addition, 38 CML-CP patients were divided into two groups: 31 treated with imatinib,7 treated with hydroxycarbamide and IFNα (see Table 1).This study also includes 25 healthy donors. Mononuclear cells were obtained by BM aspiration after obtaining informed consent. The study was approved by the Sichuan University institution review board.

**Table 1:** Patients characteristics

Patient Characteristic	n
Sex	
Male	43
Female	17
Phase	
CML-CP	38
CML-AP	9
CML-BC	13
Treatment of CML-CP	
With imatinib	31
Without imatinib	7

Abbreviations: AP: accelerated phase; BC: blast crisis;

CP: chronic phase;CML:Chronic Myelogeneous Leukemia.

### RNA isolation

Total RNA was extracted from mononuclear cells using an RNA extraction kit from Invitrogen according to the manufacturer's instruction(Carlsbad, CA, USA).RNA quality was determined by agarose gel electrophoresis and quantified spectroscopically(260 nm) using a Biophotometer (Eppendorf, Hamburg, Germany).

### Reverse-transcription PCR

Complimentary DNA was synthesized from 2 μg of total RNA from each samples using RNA PCR Kit (AMV) (Promega, Madison, WI). Commercially synthesized PCR primers were used to amplify specific Hh transcripts:

Shh(F:5'-CCTCGCTGCTGGTATGCTCGGGACT-3', R:5'-CTCTGAGTCATCAGCCTGTCCGCTC-3');Ptch1:(F:5'-GCACTACTTCAGAGACTGGCTTC-3', R:5'-AGAAAGGGAACTGGGCATACTC-3');Smo(F:5'-ACCCCGGGCTGCTGAGTGAGAAG-3', R:5'-TGGGCCCAGGCAGAGGAGACATC-3');Gli-1(F:5'-TCCTACCAGAGTCCC

AAGTTTC-3', R:5'-CCAGAATAGCCACAAAGTCCAG-3'); β-Actin(F:5'-CCAAGGCCAACCGCGAGAAGATGAC-3', R:5'-AGGGTACATGGTGGTGCCGCCAGAC-3').

The predicted sizes of the PCR products were 262 bp for Shh,395 bp for Ptch1,562 bp for Smo,391 bp for Gli-1 and 587 bp for β-Actin.PCR reaction mixtures contained 1 ul cDNA,3 ul Mgcl_2 _(25 mM),4 ul dNTP(2.5mM),10×PCR Buffer 5 ul,0.5 umol of each primer and 1.25 units of heat-stable DNA polymerase(Takara, Biotech, Japan).Amplification programmes were applied for Shh(25 cycles at 94°C,65°C and 72°C,45 s each), Ptch1(28 cycles at 94°C,30 sec;60°C,30 sec;72°C,45 s), Smo(28 cycles at 94°C,30 sec;55°C 30 sec;72°C,45 s), Gli-1(30 cycles at 94°C, 30 sec; 57°C,30 sec; 72°C,45 s). Four independent PCR reactions were carried out with different numbers of PCR cycles thus ensuring that each PCR amplification was not reach the plateau phase. Subseqently,5 ul PCR product was subjected to 1.5% agarose gel electrophoresis followed by ethidium bromide staining. The density of PCR products were measured by Bio-Rad gel imaging system(Bio-Rad, USA) of photographs of ethidium-bromide-stained agarose gels. The relative gene expression of Shh, Ptch1, Smo, Gli1 were determined by comparing the ratio of PCR products of the target cDNA segments and the β-Actin cDNA segment as a reference.

### Statistical analysis

The data are presented as means ± SEM. The differences between the mean values of two groups were evaluated by using the Student's t-test (unpaired comparison). For comparison of more than three groups, we used one-way analysis of variance (ANOVA) test followed by Tukey's multiple comparison. P values of <0.05 were considered statistically significant.

## Results

### Increased Hh target gene expression in CML

**Figure 1:**
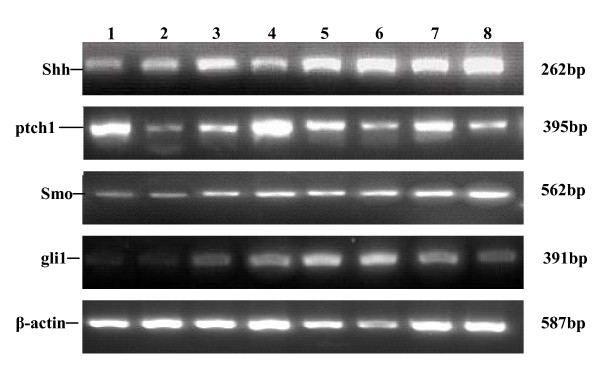
**Expression of Hh and its receptors in CML patients and normal control**. Lane 1:normal control 1:Lane 2:normal control 2:Lane 3:CML-CP case 1:Lane 4:CML-CP case 2:Lane 5:CML-AP case 1:Lane 6:CML-AP case 2:Lane7:CML-BC case 1:Lane8: CML-BC case 2.

We examined expression of Hh and its receptors in CML and normal controls by semiquantitative PCR. Shh, Ptch1, Smo, Gli1 mRNA can be detected in both CML group and normal control group. We analyzed the relative expression levels of Shh, Ptch1, Smo, Gli1 mRNA in all groups, and the results indicated that Shh, Smo, Gli1 mRNA levels in CML group were significantly higher than those in control group(p < 0.005). But there is no significant difference for the mRNA expression of Ptch1 between CML group and normal control group(p > 0.05)(see Figure 1).

### Expression of Hh and its receptors in different phases of CML

**Figure 2:**
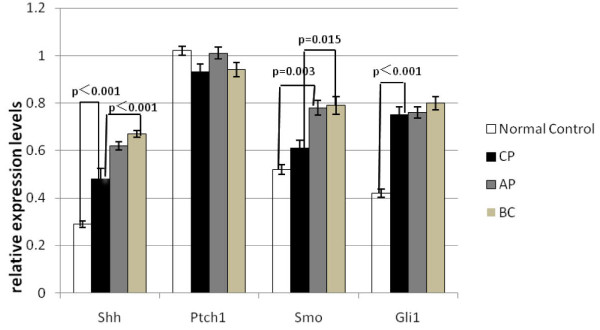
**Comparison of Hh and its receptors expression between different groups**.

Further analysis of the data revealed an association of Hh signaling activation with progression of CML. We compared the transcript levels of Hh and its receptors in patients with CML in chronic phase, accelerated phase and blast crisis. The levels of Shh mRNA in patients of CML-CP were obviously lower than that of CML-AP or CML-BC(p < 0.05), but there were no significant differences between CML-AP group and CML-BC group. Our results also demonstrated elevated Smo expression in patients of CML-BC. The relative expression levels of Smo mRNA in CML-BC group were much higher than in CML-CP group, but no significant differences were found between CML-CP and CML-AP group, CML-AP and CML-BC group. Moreover, in most of the cases, increased levels of Shh were consistent with elevated levels of Smo expression. We also found high Gli1 and Ptch1 transcripts in patients of CML-BC and CML-AP compared with the CML-CP group, but there were no significant differences between these three groups(p > 0.05)(see Figure 2).

### Expression of Hh and its receptors in CML-CP patients with IM administered or not

It is reported that expansion of BCR-ABL-positive leukemic stem cells and the maintenance of self-renewal properties in this population are dependent on intact and activated Hh signaling, therefore, it is intriguing to postulate that imatinib have no role on Hh pathway. To test this possibility, we analyzed the levels of Shh, Ptch1, Smo, and Gli1 expression in 38 CML-CP patients, with 31 patients treated with imatinib and another 7 patients treated with hydroxycarbamide and IFNα. As expected, we found that there were no significant differences of Shh, Ptch1, Smo, Gli1 mRNA expression when comparing CML-CP patients with IM treated or not(p > 0.05)(see Table 2).

**Table 2:** Expression of Hh and its receptors in CML-CP patients with IM administered or not

CML-CP	n	Expression level(°C ± S)	P value
Shh			
Without Imatinib	7	0.55 ± 0.020	0.24
With Imatinib	31	0.46 ± 0.017	
Ptch1			
Without Imatinib	7	1.21 ± 0.031	0.12
With Imatinib	31	0.87 ± 0.031	
Smo			
Without Imatinib	7	0.66 ± 0.020	0.88
With Imatinib	31	0.59 ± 0.023	
Gli1			
Without Imatinib	7	0.83 ± 0.042	0.43
With Imatinib	31	0.73 ± 0.027	

## Discussion

Hedgehog signaling pathway is important in the pathogenesis of several malignancies. Several mechanisms have been described that lead to the activation of the Hh signaling pathway in tumor cells, such as activating point mutations of Smo or inactivating point mutations in Ptch1 or SUFU [8-12]. Although inappropriate activation of the Hh signaling pathway has been shown in many cancers, the assessment of the contribution of Hh signaling pathway has not been thoroughly examined in hematologic malignancies. Given the parallels in Hh signaling between regulation of proliferation of primitive human hematopoietic cells and hematologic malignancies [13-15], we examined whether Hh signaling might also have a role in CML.

Here, with the use of semiquantitative PCR analysis, we showed that the Hh signaling components Shh, Ptch1, Smo and Gli1 were expressed in all CML patients that we screened. And the relative expression levels of Shh, Smo, and Gli1 mRNA in CML group were significantly higher than those in normal control group, suggesting that activation of the Hh pathway is quite common in CML. But the level of Ptch1 mRNA in CML and normal control group did not show significant difference. We repeated the amplification procedure several times, but there was still no difference found. The reason might be that the primary CD34^+ ^leukemic cells have been not separated. Furthermore, we found elevated Shh, Ptch1, Smo, Gli1 transcripts in advanced stages of CML, especially the levels of Shh, Smo expression were significantly higher in blast crisis than that in chronic phase of CML. A significant correlation between increased expression of both Shh and Smo in patients of CML-BC would support the hypothesis that aberrant Hh signaling contributes to CML development or progression.

The outcome for CML patients has been dramatically improved with the use of tyrosine kinase inhibitors (TKIs), leading to response rates of greater than 95% [16]. Although it is very effective in treating chronic phase CML patients, imatinib will unlikely provide a cure to these patients. Several reports indicate that discontinuation of imatinib treatment even in patients who have already achieved molecular response induces a relapse of the disease [17], and therefore, patients are forced to undergo lifelong therapy. Further studies have demonstrated that imatinib effectively eradicates Bcr-Abl-positive progenitor cells but does not target Bcr-Abl-positive CD34+ LSCs [1,2], as there is evidence that Bcr-Abl-positive LSCs remain present in the patient's bone marrow even after years of therapy and can cause relapse of disease [18-20]. Our study indicated that imatinib treatment has no significant influence on the inhibition of Hedgehog pathway of CML-CP patients.

Although responses to interferon-alpha (IFNα) are slower and less dramatic than those to imatinib, they can be durable even after discontinuation of the drug [21-23]. Unlike imatinib, the specific mechanisms responsible for IFN's clinical activity in CML are unknown. Previous report indicated that IFNα inhibits Mek phosphorylation in hedgehog pathway activated basal cell carcinoma (BCC) cells [24]. At the current time, there is still much to learn about the role of Hh signaling pathway in the development and progression of CML, and further studies will be required to understand the biological function(s) of IFNα in the Hh pathway.

In conclusion, we confirmed variable abnormalities of Hedgehog pathway activation in CML cases involved in this study, raising a possibility that combinations of ABL and Hh inhibitors might offer a new treatment strategy in CML and might help to effectively cure this disease.

## Abbreviations

AP: accelerated phase; BC: blast crisis; CML: Chronic Myelogeneous Leukemia; CP: chronic phase; Hh: Hedgehog; HSC: hematopoietic stem cell; IM: imatinib; LSCs: leukemia stem cells; PCR: polymerase chain reaction; Ptch:Patched; Shh: Sonic hedgehog; Smo: Smoothened.

## Competing interests

The authors declare that they have no competing interests.

## Authors' contributions

HZ, BL, TL and WM designed the study, BL and CZ carried out PCR, HZ, Bing Long drafted the manuscript and performed the statistical analysis. All authors read and approved the final manuscript.
